# Pathophysiology to Risk Factor and Therapeutics to Treatment Strategies on Epilepsy

**DOI:** 10.3390/brainsci14010071

**Published:** 2024-01-10

**Authors:** Ana Paula de Araújo Boleti, Pedro Henrique de Oliveira Cardoso, Breno Emanuel Farias Frihling, Luiz Filipe Ramalho Nunes de Moraes, Ellynes Amancio Correia Nunes, Lincoln Takashi Hota Mukoyama, Ellydberto Amancio Correia Nunes, Cristiano Marcelo Espinola Carvalho, Maria Lígia Rodrigues Macedo, Ludovico Migliolo

**Affiliations:** 1 S-Inova Biotech, Programa de Pós-Graduação em Biotecnologia, Universidade Católica Dom Bosco, Campo Grande 79117-900, Brazil; apboleti@yahoo.com.br (A.P.d.A.B.); cardosoliveira20@gmail.com (P.H.d.O.C.); brenoemanuelfarias@gmail.com (B.E.F.F.); bioramalho@gmail.com (L.F.R.N.d.M.); ellynesnunes@gmail.com (E.A.C.N.); rsr.lincoln.mukoyama@gmail.com (L.T.H.M.); ellydbertonunes24@gmail.com (E.A.C.N.); cristiano@ucdb.br (C.M.E.C.); 2 Laboratório de Purificação de Proteínas e Suas Funções Biológicas, Unidade de Tecnologia de Alimentos e da Saúde Pública, Universidade Federal de Mato Grosso do Sul, Campo Grande 79070-900, Brazil; ligiamacedo18@gmail.com; 3 Programa de Pós-graduação em Bioquímica, Universidade Federal do Rio Grande do Norte, Natal 59078-970, Brazil; 4 Programa de Pós-graduação em Biologia Celular e Molecular, Universidade Federal da Paraíba, João Pessoa 58051-900, Brazil

**Keywords:** epileptic syndromes, neurotransmission, comorbidities, clinical trials

## Abstract

Epilepsy represents a condition in which abnormal neuronal discharges or the hyperexcitability of neurons occur with synchronicity, presenting a significant public health challenge. Prognostic factors, such as etiology, electroencephalogram (EEG) abnormalities, the type and number of seizures before treatment, as well as the initial unsatisfactory effects of medications, are important considerations. Although there are several third-generation antiepileptic drugs currently available, their multiple side effects can negatively affect patient quality of life. The inheritance and etiology of epilepsy are complex, involving multiple underlying genetic and epigenetic mechanisms. Different neurotransmitters play crucial roles in maintaining the normal physiology of different neurons. Dysregulations in neurotransmission, due to abnormal transmitter levels or changes in their receptors, can result in seizures. In this review, we address the roles played by various neurotransmitters and their receptors in the pathophysiology of epilepsy. Furthermore, we extensively explore the neurological mechanisms involved in the development and progression of epilepsy, along with its risk factors. Furthermore, we highlight the new therapeutic targets, along with pharmacological and non-pharmacological strategies currently employed in the treatment of epileptic syndromes, including drug interventions employed in clinical trials related to epilepsy.

## 1. Introduction

Globally, epileptic disorders are generally classified into two distinct groups: common epilepsies, representing around 95% of cases, and rare epilepsies, characterizing approximately 5%. Within this last group, epileptic syndromes stand out, forming a broad and heterogeneous group of diseases that affect individuals of pediatric age [1]. Epileptic seizures are chronic in nature, characterized by the recurrent manifestation of unprovoked seizures [2]. The classification developed by the International League Against Epilepsy (ILAE) in 2017 established three distinct levels of diagnosis, including the nature of the seizure, the type of epilepsy, and the epileptic syndrome. In this context, the importance of considering the etiology and comorbidities at each diagnostic level is emphasized [3,4].

This pathology is also divided into three etiological categories: idiopathic, acquired, and cryptogenic. In childhood, the idiopathic form of epilepsy manifests itself without visible neurological signs, while the acquired form is related to identifiable structural lesions in the brain, resulting from trauma, tumors, infections, hippocampal sclerosis, as well as cerebrovascular, immunological, perinatal, and childhood disorders. In turn, cryptogenic epilepsy remains an etiological enigma, as its cause can be difficult to identify, discouraging the use of the term “cryptogenic” in modern studies due to its unclear implications [4,5].

One of the characteristics of epileptic syndromes is their pharmacological resistance, in addition to epileptic polymorphism and severe alterations in the electroencephalogram patterns [6,7]. Affected patients experience a wide range of neuropsychiatric symptoms, ranging from mild to severe, encompassing neurological impairments, mental retardation, sensory and communication deficits, and other significant changes in psychiatric, motor, and behavioral aspects [1].

An extensive combination of genetic polymorphisms, epigenetic modifications, and environmental factors, such as pollutants, diet composition, and brain injuries, emerge as key factors in the reconfiguration of brain circuits, culminating in the emergence of epileptic disorders [1,8]. Brain adaptations resulting from these factors can upset the delicate balance between excitatory and inhibitory processes. As a result, a seizure can manifest itself in different areas of the brain and propagate to other synaptically connected regions, compelling an increase in the severity of the condition [9,10]. This review provides a broad discussion of the neurological mechanisms causing the development and progression of epilepsy, as well as its risk factors, in addition to revealing the new therapeutic targets and pharmacological strategies currently used in the treatment of epilepsy syndromes. Additionally, we will also explain the drug interventions used in epilepsy-related clinical trials.

## 2. Neurological Mechanisms Underlying the Development and Progression of Epilepsy

Epilepsy is a complex neurological condition, characterized by recurrent spontaneous seizures [11]. It is a prevalent neurological disorder that affects over 50 million people [12]. Patients experience repetitive seizures, which result from abnormal, excessive, and synchronized firing of neuron groups within the brain. Seizures originating from a specific brain region are called focal or location-related, while generalized seizures occur simultaneously in both cerebral hemispheres [13]. Seizures occur when there is abnormal synchronous neuronal firing in a specific section of the brain or throughout the brain. These abnormal networks can be caused by structural, infectious, or metabolic disturbances [14]. Frequent confusion occurs between seizures and epilepsy, although these two terms are not interchangeable. The onset of a seizure is defined as either focal, generalized, unknown, or unclassifiable. The isolated occurrence of a seizure in an individual does not necessarily imply that he has epilepsy, as the seizure could have been triggered, but not be repeated. The term “epileptogenesis” encompasses the developmental trajectory that leads to status epilepticus. This concept encompasses the sequence of events that transforms the brain from a normal state to one predisposed to seizures. This transformation presupposes the hyperexcitability of neuron groups, which become prone to discharge abnormally [15].

Thus, epileptogenesis is the process of developing and expanding tissue capable of generating spontaneous seizures, leading to the development of an epileptic condition and/or the evolution of epilepsy once it is established [16]. Furthermore, epileptogenesis is connected with pervasive neuronal damage, gliosis, and microgliosis, creating an inflammatory state in the microenvironment of the neural tissue [17]. Inflammatory processes can originate in the central nervous system or be acquired from systemic circulation due to a breakdown in the blood–brain barrier (BBB) [18].

Synapses connect with all of the physical parts of an astrocyte, encompassing its terminal projections and soma. A crucial requirement for the realization and effectiveness of most of these regulatory functions is the extensive electrical and metabolic interconnection between astrocytes, facilitated by low-resistance aqueous channels known as gap junctions (GJs). This leads to the formation of large syncytium-like functional networks that overlap neuronal synaptic networks and allow their coordinated regulation and synchronization [19]. Astrocytic junctions consist primarily of the channel proteins connexin43 (Cx43) and connexin30 (Cx30), whose relative expression levels vary considerably between developmental stages and brain regions [20,21].

The progression to epilepsy is characterized by the presence of neuroinflammation, along with structural and molecular changes in the brain. These subsequent changes lead to increased neuronal hyperexcitability and a long-lasting propensity for recurrent spontaneous seizures [22]. Microglia play a role in regulating neuroinflammation and axonal sprouting and have also been reported to modulate neurogenesis. After seizures, microglia are activated and act as resident macrophages in the brain, responding quickly to injury while trying to keep physiological processes in check [23]. Changes in neuronal homeostasis are also observed, highlighting the various ways in which microglia can contribute to the development of epilepsy [22].

**Figure 1:**
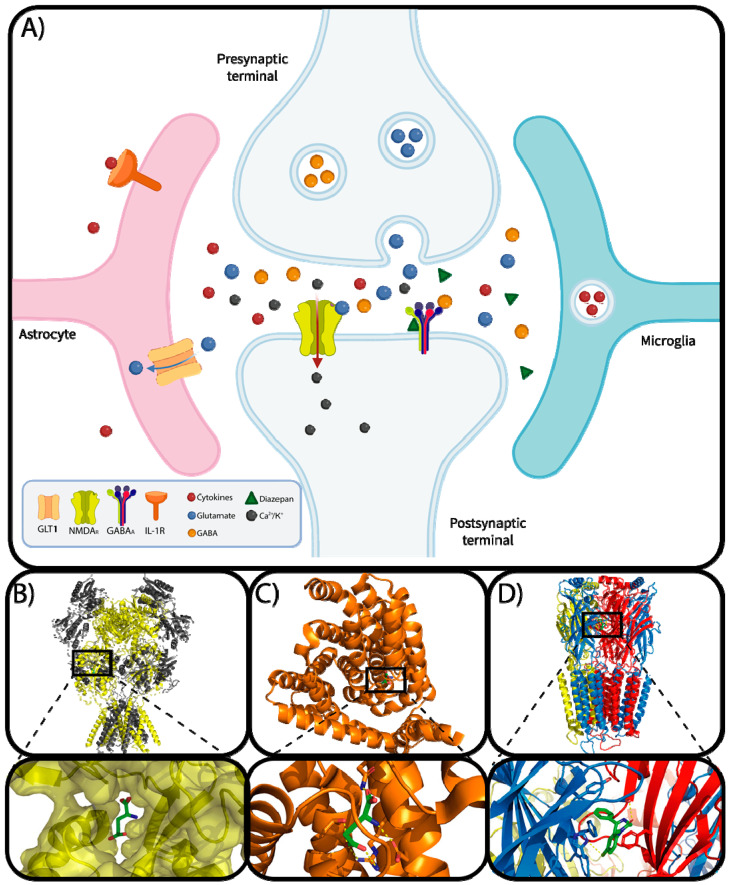
Epileptogenesisis activated by neuroinflammation and the molecular targets for the treatment of epilepsy. (**A**) Synaptic cleft demonstrating a scenario of neuroinflammation influencing epileptogenesis. Due to the initial mechanisms of inflammation (breakdown of the blood–brain barrier and/or genetic factors), there is an expression of cytokines by the microglia, directly influencing glutamate reuptake activity. Furthermore, astrocytes, which naturally contribute to CNS homeostasis, can promote the maintenance of epilepsy by causing an imbalance of glutamate in the synaptic cleft. (**B**) NMDAR transmembrane protein focusing on the glutamate binding site. In a normal situation, this receptor is responsible for transporting ions to the cellular interior of the postsynaptic neurons. In an epilepsy situation, glutamate levels are elevated, promoting greater activation of this receptor, resulting in hyperexcitability. (**C**) In the astrocytes, GLT-1 transports protein in combination with glutamate, focusing on the binding site. Naturally, this protein acts in the reuptake of glutamate, and in the presence of neuroinflammation, the cytokines released by the microglia through the activation of IL-1R promote the reduction of GLT-1 expression, increasing glutamate levels in the synaptic cleft. (**D**) GABAA transmembrane protein in the diazepam complex, focusing on the binding site. In a normal situation, this receptor is responsible for the uptake of GABA, resulting in a suppression of neuronal activity. In the presence of diazepam, this receptor has an increased affinity for GABA, reducing epileptic effects.

After seizures, cytokines such as IL-1β, IL-6, and TNF-α are released, modulating inflammatory responses in the brain. Studies indicate that these cytokines influence NMDA receptors, synaptic plasticity, GABAergic neurotransmission, and neuronal excitability, contributing to the development and recurrence of seizures. Inhibiting IL-1β activity, for example, has shown a reduction in seizures in rodent models. TNF-α, released by microglia and astrocytes, positively regulates synapses, glutamate release, and GABA levels. Furthermore, IL-6, regulated by TNF-α and IL-1β, negatively influences neurogenesis in the hippocampus and increases microgliosis, possibly contributing to epileptogenesis. (Figure 1A) [17,18,24,25]. Prostaglandins activate the EP3 receptor on astrocytes, leading to increased glutamate release and inducing hyperexcitability and neuronal cell death. Conversely, inhibiting the EP3 receptor may delay the onset of seizures [26].

In addition to epileptogenesis, studies regarding genetic and lesion-induced epilepsies also indicate common pathologic mechanisms. The proposal of an imbalance between excitation and inhibition has been considered as a mechanism of ictogenesis and epileptogenesis. This imbalance is associated with an increase in extracellular glutamate in the brain and/or a decrease in GABA concentrations, resulting in excitotoxicity, convulsions, and cell death [27]. The implication of an imbalance between excitation and inhibition highlights the importance of understanding the relationship between epilepsy and glutamate, a crucial neurotransmitter in the central nervous system and the most abundant amino acid in the mammalian brain. Glutamate plays an essential role in several processes, such as learning, memory, cognition, and emotion, and all activities related to glutamate in the brain occur in the extracellular space [28,29].

Glutamate is released by glutamatergic neurons into the extracellular space, acting on the ionotropic and metabotropic receptors. Neuronal and astrocytic transporters prevent overexcitation by removing glutamate from the synaptic cleft. In the astrocytes, glutamate is converted to glutamine, transported back to the neurons, and reverted to glutamate, allowing for continued release. Glutamate regulation involves both the neurons and astrocytes, and any dysfunction in the system can cause an imbalance between excitation and inhibition [28,30]. Specifically, astrocytes may play a role in the pathogenesis and pathophysiology of epilepsy by modulating synaptic transmission through the release of glycotransmitters such as glutamate, ATP, and D-serine, thus contributing to homeostatic control [19,20]. Astrocytes also contribute to the supply of glutamine to the GABAergic neurons, converting it into glutamate and subsequently, into gamma-aminobutyric acid (GABA) through the action of glutamate decarboxylase. This GABA is then packaged into vesicles for release. GABA is the main inhibitory neurotransmitter, and it is crucial to maintain an adequate balance between it and glutamate. An imbalance between excessive glutamate and/or inadequate GABA can result in overexcitation of the central nervous system, predisposing the occurrence of seizures [29,31].

Dysregulation in the glutamatergic mechanisms in epilepsy involves dysfunctions in the interactions between the neurons, the astrocytes, or both of these. This may include dysregulation of the ionotropic or metabotropic receptors, abnormal expression of the astrocytic glutamate transporters, and the malfunction of the neuronal or astrocytic enzymes. Genetic mutations in the NMDA receptors, such as GRIN1, GRIN2B, and GRIN2D, and mutations in the AMPA receptors, which increase AMPA expression, are suspected of contributing to physiological imbalances in epilepsy, remodeling and reconfiguring the neural networks. Glutamatergic dysregulation can lead to the accumulation of glutamate in the synapse, as well as the overactivation of glutamate receptors, resulting in excitotoxicity and eventually, cell death [32,33].

## 3. Genetic Influence on Epilepsy

Currently, cases of severe epilepsy are closely related to genetic factors. About 0.4% of the human population have genetic epilepsy; this percentage corresponds to about 30% of all known epilepsies. More than 50 genes associated with this pathology have recently been identified [34]. It is known that the main genetic influence on severe epileptic conditions is related to mutations in the ion channel neurotransmitters, causing neuronal hyperexcitability or exhaustion of the inhibitory mechanisms, which results in seizures. However, other genes, such as those that cause mutations in transcription factors, intracellular signaling molecules, chromatin remodelers, metabolic enzymes, and even genes in the mitochondrial complex [34], have been identified in individuals with genetic epilepsy.

One of the most important identified mutations is in the existence of R-type calcium channels. Individuals exhibiting the CACNA1E variant present early epilepsy, which is characterized by presenting not only seizures, but also macrocephaly, developmental delay, severe hypotonia, congenital contractures, hyperkinetic movements, and early death. This type of epilepsy is known as early infantile epileptic encephalopathy [34,35].

In addition to GABRB3-type mutations, an apparent connection with early childhood epileptic disorders is also observed, accompanied by significant impairment in the intellectual development of those affected. Additionally, mutations in the GRIN2A and GRIN2B genes, related to the NMDA receptors, can trigger epileptic disorders in neurodevelopment, epilepsy spectrum disorders, and idiopathic focal epilepsy. The GRIN1 gene, in turn, is associated with cases of epilepsy accompanied by developmental delay, along with hyperkinetic movement disorders and infantile hypotonia [36]. The KCNA2 gene was recently identified as one of the genes responsible for epilepsy. KCNA2 is related to the current-dependent potassium channel, which includes four subunits that can assume different conformations, and which is expressed in the central nervous system [37]. Studies have shown that a mutation in KCNA2 can cause dramatic loss or gain of function. Studies with mice show that individuals in whom this gene was deleted were more likely to develop seizures. In addition, some mutations were observed in humans, such as Q213* and G398C, which caused loss of function, as well as mutation L298F, which affects the second arginine of the amino acid sequence and causes a 13-fold increase in channel current, and E157C, which showed an up to 5-fold increase in current [37].

A newly discovered gene related to the onset of focal epilepsy, DEPDC5, was also shown to be associated with other epileptic syndromes, and it is related to the cases of childhood epilepsy accompanied by sudden death. Although it is not included in the group of epilepsies that cause impairment in intellectual development, some individuals with this variant may present with intellectual impairment and the development of ASD [38]. Another study showed that patients with developmental encephalopathy and epilepsy exhibited groups of genes that were previously unrelated to epilepsy, including FGF12, GABBR1, GABBR2, ITPA, KAT6A, PTPN23, RHOBTB2, and SATB2 genes [39].

## 4. New Therapeutic Targets and Pharmacological Strategies

Studies related to the development of new anti-epilepsy drugs have been increasingly requested due to the recent emergence of resistance to anti-epilepsy drugs currently on the market, as well as scientific evidence that proves the long-term ineffectiveness of the use of anti-epilepsy drugs [40]. About one-third of people with epilepsy disorders exhibit incomplete or partial control of seizures, even with the administration of anticonvulsant drugs, used in combination or alone [40,41]. Over the past few years, a variety of therapeutic targets have been elucidated to develop anti-epileptic drugs. For a drug to be considered an anticonvulsant, it must promote a balance between the excitation and inhibition of the neurotransmitters, primarily including the GABA and glutamate pathways [42,43]. As the targets of their main mechanisms of action, molecular agents act directly on the physiological process of the designated neuronal element, such as ion channels, enzymes, transport proteins, receptors, or even agents that regulate gene expression, allowing for a partial or total reduction of symptoms observed in an epilepsy condition [42,44].

According to the Epilepsy Foundation (https://www.epilepsy.com/ (accessed on 10 July 2023)) and DailyMed (https://dailymed.nlm.nih.gov/dailymed/ (accessed on 10 July 2023)), in regards to the current commercially available drugs for the treatment of seizures, there are 36 antiepileptic drugs (AEDs) approved by the FDA (Supplementary Table S1). Their mechanisms of action are mostly unknown, but some drugs exhibit more clearly elucidated or proposed mechanisms of action; the most common treatment targets are the GABA system, the voltage-gated channels, the synaptic vesicle protein 2A, the α-amino-3-hydroxy-5-methyl-4-isoxazolepropionic acid (AMPA) glutamate receptor, and the N-methyl-D-aspartate (NMDA) receptor. Examples of currently marketed AEDs include benzodiazepines, carbamazepine, and levetiracetam [45,46,47]. While most anticonvulsant medications demonstrate effects related to controlling and reducing symptoms, they do not necessarily act specifically by modulating pathogenic disease mechanisms, as the majority of cases are still not fully elucidated in regards to their pathogenesis [48].

However, symptom reduction is noted for different types of treatment, according to the observed pathology, with monotherapy methods using only one type of medication, and polytherapy techniques targeting different therapeutic targets, altering pharmacological doses, or alternating active ingredients derived from the same class of action [49,50,51]. Due to the complexity of the disease and its multiple variants, it is necessary to search for new therapeutic targets in order to develop innovative pharmacological strategies, as the same drug may have different effects, depending on the patient [52,53,54].

Over the last few years, epigenetics has greatly contributed to advancement in the understanding of epileptogenesis and epilepsy syndromes, demonstrating that there is a wide range of alteration in the expression of genes related to cellular physiology, including neural structure assembly, as well as the abundance of neurotransmitters and ion channels that play a critical role in the nervous system [55]. Furthermore, studies reveal the role of neuroinflammation as a process that can not only favor, but also intensify epileptogenesis; when seizures occur, the brain releases a series of chemical mediators, mainly glutamate, causing neuronal hyperexcitability (Figure 1A) [33,54,56]. The key chemical mediators of neuroinflammation resulting from a seizure event include the activation of pathways such as the TLR, IL-1, and IL-6 receptors, the prostaglandin-arachidonic acid cascade, oxidative stress, TNF, and caspase 1 [57,58,59,60,61].

Neuroinflammation, among other epileptogenesis-associated pathways, can lead to neurophysiological changes in the tissues, including neuronal dysfunction, stress, and acute or chronic neurodegeneration [62,63]. In recent years, the neuroinflammation pathway has been isolated as an emerging target for the development of new drugs with anti-neuroinflammatory potential for the treatment of epilepsy [64]. These pathways may be even more promising, as they act directly on the disease mechanism, and not merely by fighting symptoms [65]. Neuroinflammation can be considered one of the most relevant causes for the emergence of this phenomenon, and this occurs mainly due to the influence of cytokines released by microglia that directly interfere with the function of other glial cells [66,67].

When a neural stimulus is released in a normal state, there is a balance between the excitatory and suppressive neurotransmitters, such as glutamate and GABA, which bind to their respective postsynaptic neuroreceptors, such as NMDA_R_ (Figure 1B) and GABA_A_ [68]. Furthermore, the presence of glial cells such as astrocytes plays an important role in glutamate reuptake [69]. In a neuroinflammation scenario, the presence of cytokines in the surroundings of the synaptic cleft can activate astrocyte membrane receptors, triggering a series of intracellular routes that result in the production of factors that influence the expression of GLT-1, a transporter protein that acts in the reuptake of glutamate, keeping the levels of this neurotransmitter stable (Figure 1C) [70,71]. Without the action of this agent, the synaptic cleft contains more glutamate than GABA, resulting in a greater interaction with the excitatory neuroreceptors, causing the phenomenon of frequent seizures [72,73].

Inhibiting microglial activity using anti-inflammatory drugs may directly impact the function of regulating glutamate levels mediated by the astrocytes, as GLT-1 can remain functional [74]. This protein is made up of three identical subunits that join together, forming a complex capable of transporting up to three glutamate units [75]. Just as external factors, such as LPS, can activate microglia, the seizure event itself can prolong the effect of neuroinflammation, due to exacerbated levels of excitatory neurotransmitters and pH changes in the brain, causing injuries that lead to a cycle of chronic neuroinflammation, progressively increasing the severity of the seizures [76].

Among the existing medications, diazepam, a drug belonging to the benzodiazepine class, is well characterized, capable of quickly crossing the blood–brain barrier and interacting with GABAergic receptors, increasing its affinity for γ-aminobutyric acid, promoting a relaxation and suppression of motor neuronal action (Figure 1D) [77,78]. The GABA_A_ neuroreceptor is an important target for the development of drugs exhibiting neuronal action, as it possesses different allosteric sites which interact with compounds with analgesic, sedative, antispasmodic, anxiolytic, and anesthetic properties; it is a dimer composed of five different subunits—α1-A, α1-D, β3-B, β3-E, and γ2-C—interconnected together, forming a transmembrane ion channel which is present in the postsynaptic region of the neurons [79].

In addition to neurons, other cellular targets responsible for the maintenance and homeostasis of nervous tissue, such as microglia, a resident macrophage of the central nervous system that plays a regulatory and protective role under physiological conditions, may be promising targets for the development of anti-neuroinflammatory drugs [80]. Several studies focusing on the development of biopharmaceuticals with the potential to inhibit microglia are currently being carried out; for example, antimicrobial peptides with anti-inflammatory action are being investigated [81]. Ca-MAP1 is a multifunctional synthetic peptide with inhibitory action on microglia stimulated by LPS, rationally designed for use in an *in vitro* neuroinflammation model using BV-2 cell culture [82].

Peptides are molecules that may have a high potential for the treatment and prevention of epileptogenesis, as they are easily absorbed by the blood–brain barrier due to their small size, and they interact with specific targets, promoting a neuroprotective effect [83]. Arginine and lysine-rich cationic peptides exhibit a range of biological activities, including immunomodulation, the blockade of ion channels, and tropism of membranes with lytic or cell penetration action, contributing to potential permeability and access to the central nervous system [84,85,86,87]. Some peptides, such as TAT-NR2B9c, CN-105, and RD2, have a neuroprotective function and can be used for the treatment of ischemic stroke, hemorrhagic stroke, and Alzheimer’s disease. Based on clinical data, cationic arginine-rich peptides have been demonstrated to be safe when administered in therapeutic doses by slow venous infusion [88].

In general, all types of epilepsy are related to increased extracellular levels of Ca^2+^ ions and glutamate, contributing to the hyperpolarization and hyperexcitability of motor neurons. Cannabidiol (CBD) is a compound derived from *Cannabis sativa*, and it has an intracellular mechanism of action that acts directly on receptors such as GPR55 and TRPV1, which play a key role in epileptogenesis, allowing for low levels of membrane polarization in the neurons, in addition to blocking the reuptake of adenosine, promoting an increase in extracellular levels of adenosine in the nervous system [89].

Finally, more recent studies have demonstrated that the cytoskeleton and structuring proteins that promote the attachment and formation of synaptic clefts may be crucial factors that effectively contribute to the progression of the epileptic phenomena [90]. This occurs because Arc proteins are responsible for the formation of neuron projections, which interfere in the period of cell–cell interaction, as well as in the neuroplasticity of cells in the sclerotic hippocampus, a neuronal area that demonstrates relationships with cognitive activity [91,92].

Changes in the expression of Arc protein can trigger greater neuronal plasticity during epileptic seizures, leading to an increase in the formation of new connections between the neurons and promoting a chance of electrochemical imbalance in the neuronal tissue [90]. Furthermore, the upregulation of cytoskeletal protein (Arc) mRNA, related to activity in the dentate granule cells (DGCs) of the sclerotic hippocampus, may be a crucial molecular target in the development of new therapeutic strategies [90,92]. These factors, among the others mentioned above, contribute to epileptogenesis and the emergence of specific lesions in the tissue, resulting in an inflamed region, with impact on the microglia [93].

Although our understanding of the seizure phenomenon is still not completely clear, several molecular targets can be used for the development of new drugs, including regulatory agents of gene expression, i.e., GABAergic, purinergic, or opioid neuroreceptors, which promote the suppression or reduction of seizure neuronal activity [36]. Targets involved in neuroinflammation include the inhibition of the microglia and pro-inflammatory receptors, in addition to blocking the ions involved in neuronal membrane polarization [94].

## 5. Drug Interventions in Epilepsy-Related Clinical Trials

In the search for new forms of treatment or the reuse of existing drugs for the treatment of many conditions and illnesses, including epilepsy, clinical trial research is a method for ensuring proper drug dosage, efficacy, and safety. A clinical trial comprises many phases, with each one designed to investigate one aspect of drug usage; it is divided into preclinical and phases 0 to IV [95,96]. Within the research regarding new drug treatments for epilepsy and epilepsy-related conditions, we identified 92 clinical trials, completed since 2013, in the platform Clinical Trials (https://www.clinicaltrials.gov/ (accessed on 10 July 2023)) found in the National Center for Biotechnology Information database (Table 1). 

### 5.1. Lacosamide

One well-studied drug for epilepsy is lacosamide, a third-generation antiseizure drug for partial-onset seizures [97] that has been used in 2206 participants enrolled in 10 clinical trials for epilepsy, with or without partial-onset seizures. The lacosamide mechanism of action is predominantly exerted by a slow selective sodium channel inactivation, as it may bind to the collapsin response mediator protein-2 [98,99].The experimental use of lacosamide in clinical trials may be employed using two administration pathways, either oral or intravenous, and the drug concentration ranges from 2 mg·(kg·day^−1^)^−1^ to 12 mg·(kg·day^−1^)^−1^ in oral solution intake; 50 mg·day^−1^ to 600 mg·day^−1^ for tablet intake; and 20 mL of 10 mg·mL^−1^ of lacosamide for intravenous application.

The clinical trial study results using lacosamide showed a wide efficacy in the reduction in seizures or the achievement of “seizure-free” days or months; with a large number of participants, the efficacy rate can vary, depending on the number of participants and their ages. The two clinical trials with the greatest number of participants, as identified with the National Clinal Trial (NCT), are represented as NCT01964560 and NCT01832038, with 540 and 473 participants, respectively. 

The NCT01964560 study design included male and female participants, with a mean age of 7.4 ± 5.4 years, mostly of white ethnicity, and reported that 537 participants experienced a mean percentage of 66.96 ± 36.18 seizure-free days within the 96 weeks of the study. The NCT01832038 study enrollment involved male and female participants with a mean age of 32.7 ± 12.0 years, mainly Chinese, demonstrating that 57.1% of the 471 participants described a ≥50% reduction in partial-onset seizure frequency from baseline per 28 days. The clinical trials NCT01964560 and NCT01832038 have also reported serious and non-serious adverse events in 77.2 and 86.7% of all its participants, respectively. Those adverse events may or may not be lacosamide treatment-related; the most frequent non-epilepsy-related adverse events include vomiting, diarrhea, pyrexia convulsion, pneumonia, nausea, upper respiratory tract infection, nasopharyngitis, blurred vision, abdominal pain, and many others.

### 5.2. Cannabidiol (CBD)

One emerging drug used in the treatment of epilepsy is GWP42003-P, also known as CBD [89]; it has been tested on 1604 participants enrollment over 13 clinical trials over the last decade. Clinical trials studying the epileptic condition also include other epilepsy-related conditions, such as infantile spasms, Sturge–Weber syndrome, Dravet syndrome, seizures, and Lennox–Gastaut syndrome. The CBD antiepileptic mechanism of action is unknown; however, it is proposed that it can act on multiple molecular targets due to its high affinity to the transient receptor potential vanilloid-1 (TRPV1), the desensitizing cation channel, and other ion channels. Another possible molecular target for the CDB anticonvulsant mechanism of action is the equilibrative nucleoside transporter-1 (ENT-1) and its interactivity with the purinergic system or the carrier of Ca^2+^ ion, called the 55-receptor, coupled to G-protein (GPR55) [89,100].

In epilepsy-related clinical trials, the CBD administration route is oral, with concentrations ranging from 2 mg·(kg·day^−1^)^−1^ to 40 mg·(kg·day^−1^)^−1^. The three largest CBD clinical trials are NCT02224573, NCT02224560, and NCT02224703, with 681, 225, and 199 patients, respectively. These three trials are focused on the condition of epilepsy related to Dravet syndrome and/or Lennox–Gastaut syndrome. The clinical trial NCT02224573 includes a male and female enrolment, with 315 participants with Dravet syndrome and 366 with Lennox–Gastaut syndrome; the participant’s mean age is 9.7 ± 4.4 and 15.9 ± 9.5 years, respectively. The efficacy of CDB treatment for epilepsy has shown that 52.6 and 51.4% of the participants with Dravet syndrome and Lennox–Gastaut syndrome, respectively, have reported a ≥ 50% reduction in total seizures in the final 12 weeks of the clinical trial.

Participants in the clinical trials NCT02700412 and NCT02695537, with a focus on the CBD treatment of epilepsy and seizure conditions, demonstrated that in children and adults, there is a significant reduction in seizure severity, frequency, or both [101]. The most common adverse effects found in the participants in CDB clinical trials were convulsion, diarrhea, pyrexia, decreased appetite, somnolence, and pneumonia [102,103].

### 5.3. Perampanel

One broad-spectrum antiepileptic drug is perampanel, an FDA-approved monotherapy orally active, non-competitive, selective α-amino-3-hydroxy-5-methyl-4-isoxazolepropionic acid (AMPA) receptor antagonist. Its antiepileptic mechanism of action includes the reduction of stimuli in the AMPA receptors via the AMPA receptor antagonism, thus exerting an anticonvulsant effect, inhibiting seizure generation and dissemination [104,105,106]. In perampanel clinical trials 334 participants were enrolled in five clinical trials, with a focus on the conditions of epilepsy, seizures, and partial-onset or primary generalized tonic–clonic seizures. The dosage range for perampanel is 2 mg·day^−1^ to a maximum of 16 mg·day^−1^, administered either orally or intravenously, with the largest clinical trial using perampanel (NCT02849626) utilizing a starting maximum dosage of 8 mg·day^−1^, which could be increased, if tolerated. For participants taking any enzyme-inducing antiepileptic drugs (EIAEDs), the adjunctive dose of perampanel was 12 mg·day^−1^ maximum [107].

The clinical trial NCT02849626 enrolled 180 male and female participants, with a mean age of 8.1 ± 2.0 years, and resulted in a 40% reduction in focal seizures (FS), a 59% reduction in focal to bilateral tonic–clonic seizures (FBTCS), and a 69% reduction in generalized tonic–clonic seizures with the adjunctive treatment with perampanel. The most common treatment-emergent adverse events were somnolence, nasopharyngitis, dizziness, irritability, pyrexia, and vomiting [107,108]. Another larger perampanel clinical trial is the NCT02726074 trial, which focuses on monotherapy for epilepsy, with 106 male and female enrolled participants, with a mean age of 42.2 ± 14.2 years, receiving a starting dose of 2 mg·day^−1^, increasing by 2 mg·day^−1^ every 2 weeks, if necessary, to a maximum dose of 12 mg·day^−1^. The treatment response was a 100%reduction in the responder rate in 47 and 75% of participants with partial onset seizures, with or without secondary generalization and secondary generalized tonic–clonic seizures, respectively.

### 5.4. TAK-935

A novel compound TAK-935, also known as soticlestat, is an inhibitor of cholesterol 24-hydroxylase (CH24H), a brain-specific cytochrome P450 family enzyme essential for the homeostasis of brain cholesterol. TAK-935 is capable of restoring the excitatory/inhibitory balance in many preclinical hyperexcitability models. TAK-935 was tested in 230 enrolled participants in five clinical trials over the last decade [109,110]. The TAK-935 clinical trial was focused on the conditions of epilepsy, Dravet syndrome, Lennox–Gastaut syndrome, 15q duplication syndrome, CDKL5 deficiency disease, and developmental and/or epileptic encephalopathies. The administration pathway of TAK-935 is oral or by PEG tube/G-tube; the concentration can range from 50 to 600 mg·day^−1^.

The largest clinical trial was NCT03650452, which included 141 mostly white or Asian male and female participants, with a mean age of 9.5 ± 4.0 years. The clinical trial NCT03650452 applied TAK-935 orally or via PEG tube/G-tube, with a dosage of 200 mg/day, followed by 400 mg/day, then 600 mg/day, up to week 20. This treatment resulted in a reduction in seizure frequency of 27.76, 36.50, and 18.46% in epilepsy, Dravet syndrome, and Lennox–Gastaut syndrome, respectively. The most common treatment-emergent adverse events involving TAK-935 were upper respiratory tract infection, pyrexia, nasopharyngitis, decreased appetite, vomiting, somnolence, diarrhea, lethargy, fatigue, pneumonia, irritability, and constipation [111].

### 5.5. Ganaxolone

Another noteworthy drug is ganaxolone, an FDA-approved first-in-class medication used to treat seizures in patients with cyclin-dependent kinase-like 5 (CDKL5) deficiency disorder [112,113]. Its mechanism of action is thought to be the modulation of the synaptic and extrasynaptic GABA_A_ receptors through binding in the allosteric sites of the receptor. This causes a hyperpolarization of the neuron and an inhibitor effect on neurotransmission, reducing the chance of a successful potential depolarization [114,115]. Ganaxolone has been employed in clinical trials, with the enrollment of 605 participants in six clinical trials, with a focus on drug-resistant partial onset seizures, CDKL5 deficiency disorder, tuberous sclerosis, PCDH19-related epilepsy, status epilepticus, convulsive status epilepticus, non-convulsive status epilepticus, and epilepsy.

The dosage applied in clinical trials of ganaxolone depended on the administration route, with the intravenous application concentration of ganaxolone ranging from 500 to 1800 mg·day^−1^. The clinical trial NCT01963208 was the largest trial employing ganaxolone, with 405 mostly white male and female participants, with a mean age of 39.7 ± 11.7 years. The clinical trial NCT01963208 yielded a result of a 21.28% reduction in seizure frequency from baseline to week 14 of the study; within the same clinical trial in the same period, 28.1% of the participants treated with ganaxolone reported at least a 50% decrease in 28-day seizure frequency. The most common adverse events observed with ganaxolone treatment are fatigue, nasopharyngitis, somnolence, dizziness, and headache.

### 5.6. Everolimus

Two clinical trials used everolimus for seizure control, NCT02451696 and NCT01713946, with 14 and 366 participants, respectively. The trial NCT02451696 was conducted employing participants with TSC and refractory epilepsy. Four participants were approved for treatment with everolimus for 7–28 days, at a concentration of 4.5 mg·L^−1^ orally, yielding plasmatic concentrations of 5 to 15 ng·mL^−1^. The participants who received treatment had a mean age of 18.25 ± 10.1 years, while the control group had a mean age of 13.1 ± 12.3 years. In study NCT02451696, the presence of adverse effects, which were not reported, and levels of vascular endothelial growth factor (VEGF), mTOR brain tissue-s6 phosphate obtained by Western blotting, and HMGB1 expression in brain tissue were investigated. These methods noted a difference in phospho-S6 expression, which is mainly associated with seizure cases. On the other hand, study NCT01713946 was carried out with three different groups, receiving concentrations of 3 to 7 ng·mL^−1^, 9 to 15 ng·mL^−1^, and a placebo group, employing dispersible tablets for oral suspension. The age of the participants ranged from 6–65 years old, and the study lasted for 12 weeks. A total of 24.8% of the participants receiving the lowest dose, and 42% of participants in the highest dose group showed a 50% decrease in seizure frequency [116].

### 5.7. Ataluren

Another drug used as a possible treatment for seizures involving Dravet syndrome and CDKL5 deficiency disorder is ataluren, a drug whose main action is for preterm infants, allowing for the reading of the ribosome of an mRNA with the para codon generated in a full-length protein [117]. In the clinical study NCT02758626, ataluren was used to control seizures arising from the aforementioned diseases. There were seven children with DS and eight with CDKL5 deficiency disorder included in the study, with a mean age of 6.4 and 3.5 years, respectively, ranging from 2 to 11 years old. The study was a double-blind crossover with the use of a placebo as a control; the participants were authorized to receive doses of 10 mg·kg^−1^ in the morning, and 20 mg·kg^−1^ in the middle of the day, in the form of powder for suspension. However, the drug did not demonstrate efficacy in controlling the seizures. The treated groups did not show any significant difference compared to the placebo group. The drug had some adverse effects, but none were of high severity.

### 5.8. Levetiracetam (LVT) and Valproic Acid (AVP)

Studies were conducted to compare the tolerability and efficacy of anticonvulsant drugs with different mechanisms of action: levetiracetam and valproic acid. Levetiracetam is a drug that binds to the SV2A protein, preventing the release of glutamate in the neurons, demonstrating its antiepileptic action [118,119]. On the other hand, valproic acid is also an antiepileptic drug, which acts by inhibiting succinyl semialdehyde dehydrogenase, increasing the concentration of succinyl semialdehyde, an inhibitor of GABA transaminase, thereby increasing the concentration of the neurotransmitter GABA [120].

The clinical study NCT03940326 used both drugs in the treatment of 103 participants with idiopathic generalized epilepsy. The participants were divided into two groups: one treated with levetiracetam (LVT) and the other with valproic acid (AVP). The groups had a mean age of 26.2 ± 8.1 and 29 ± 9.7, respectively. Levetiracetam doses started at 500 mg/week and increased to a dose of 200 mg per day, with further increases to 300 mg per day in cases of seizures. Valproic acid was also started at 500 mg per week, with a maximum dose of 1500 mg·day^−1^, increased to 2000 mg·day^−1^ in cases of seizures.

As a result, the patients in the LVT group experienced their first seizures, on average, after 169 ± 6.1 days, while the AVP group experienced their first seizures after an average of 178 ± 2.2 days. Regarding the absence of seizures, 88.9% of participants in the LVT group and 86.2% in the AVP group did not experience seizure episodes. In the LVT group, 8.9% of the participants discontinued the medication, while in the AVP group, 10.3% did so, with an average time of 220 ± 8.7 and 172 ± 4.1 days, respectively. Some adverse events were observed, and valproic acid showed a higher rate of side effects, mainly in terms of weight gain, which was reported by 27.59% of the participants. In addition to this study, others were conducted using different drugs, but involving LVT as one of the tested drugs. The studies NCT02201251, NCT01982812, NCT02707965, and NCT03695094 employed fewer than 70 participants. However, study NCT01954121, comprising 436 participants, compared LVT and carbamazepine (CBZ), a medicine that modulates synaptic transmission and which is also used as an antiepileptic [121].

### 5.9. Brivaracetam

Another drug from the class of SV2A protein vesicle inhibitors is brivaracetam. Two studies, NCT04882540 and NCT03405714, evaluated the pharmacokinetics of the drug. The latter specifically included participants with epilepsy who were older than 1 month but younger than 16 years of age. The third study, NCT03021018, involved 46 participants with a mean age of 42.12 ± 13.06 years. The drug provided seizure control within 6, 8, and 12 h, without presenting serious adverse effects to the patients.

In the study group NCT01954121, the participants were divided into two groups: one treated with LVT (218 participants) and the other with CBZ (215 participants). The mean age of the LVT group was 37.8 ± 16.2 years, while the CBZ group had a mean age of 33.3 ± 14.3 years. The first group received LVT at a dose of 250 mg twice daily for 2 weeks, followed by evaluation over the next 27 weeks after stabilization. In the CBZ group, the starting dose was 200 mg once a day, and the participants were also evaluated for 27 weeks after stabilization. Over 6 months, the participants in both groups were analyzed for the absence of seizures. Out of the 186 participants in the LVT group, 47.3% of them remained seizure-free during this period. In the CBZ group, which consisted of 171 participants, 68.4% remained seizure-free. The most common adverse effect observed during the study was nasopharyngitis, a non-serious adverse effect, reported by 42.20% of the LVT group and 43.26% of the CBZ group.

### 5.10. Benzodiazepines

Benzodiazepines are a class of drugs widely used to treat epilepsy or seizure episodes. These drugs act as agonists of the inhibitory action of GABA, causing hyperpolarization and stabilization of the neuronal membrane, resulting in antiepileptic effects [122]. Benzodiazepines are divided into subclasses based on their duration of action. Alprazolam (APZ) shows fast action and high potency, while diazepam (DZP) exhibits a long duration and medium potency [122].

Clinical studies were conducted using DZP to explore new routes of administration for the medication. Out of four studies, three evaluated new routes of administration. Studies NCT03222349, NCT03179891, and NCT03953820 focused on these new routes, while study NCT03428360 assessed the safety and tolerability of employing DZP buccal film. Other studies involving APZ were NCT02351115, which evaluated the photosensitivity in participants using the drug, and NCT03478982, which involved 156 participants and aimed to determine the pharmacokinetics of seizure episodes using staccato alprazolam administered via oral inhalation. Another benzodiazepine investigated was clobazam (CBZ). Trials were conducted to understand its interactions with CBD. The NCT02564952 trial showed that out of 18 participants, 4 experienced adverse events from this drug interaction. However, in the NCT02565108 trial, which involved 20 participants, no such interaction was noted.

### 5.11. Lamotrigine (LMT)

Another class of medication that inhibits neural excitability is lamotrigine (LMT). It inhibits the calcium channels, which are linked to the release of neurotransmitters such as glutamate and aspartate [123]. The studies NCT02100644 and NCT02404168 focused on understanding the drug’s pharmacokinetics and its interaction with other drugs. In the study NCT02404168, LMT was used in four participants with epilepsy, aged 22–68 years. The study aimed to assess the parameters of the area under the curve and maximum concentration (CMAX) for both reference and generic LMT, which are important constants in drug bioequivalence studies. The comparative CMAX between the drugs showed 8836 ng/mL for the reference and 9024 ng·mL^−1^ for the generic LMT. Among the adverse effects reported by patients, headache was reported by three out of four participants.

On the other hand, study NCT02100644 involved 33 participants with a mean age of 25.6 ± 7.73). They underwent LMT treatments with AVP, receiving different doses which were divided into escalation, reduction, and maintenance phases. The concentration of AVP ranged from 400 to 1200 mg·day^−1^ during the escalation phase, and LMT gradually escalated from 25 mg·day^−1^. In the maintenance phase, AVP was fixed at 300 mg·day^−1^, and the LMT concentration was set at 200 mg·day^−1^. In the reduction phase, the AVP dose was reduced to 200 mg·day^−1^, and the LMT dose to 100 mg·day^−1^, which could be adjusted in case of seizures occurring during the day. The results showed that during the maintenance phase, the participants were evaluated according to the number of days with seizures. They experienced seizures on only two days during a monitored period of 46 weeks. None of the participants reported serious adverse reactions, but 69.7% reported non-serious adverse reactions.

### 5.12. Pregabalin

Pregabalin is a drug that acts by binding to α2δ voltage-activated calcium channels, which inhibits the release of excitatory neurotransmitters [124]. This drug has been studied for the treatment of seizures and epilepsy. The trial NCT01747915 was conducted with 219 participants, with a mean age of 25.2 ± 13.1 years, and the participants were divided into three different treatment groups: 5 mg·(kg·day^−1^)^−1^, 7 mg·(kg·day^−1^)^−1^, or 300 mg·day^−1^; 10 mg·(kg·day^−1^)^−1^, 14 mg·(kg·day^−1^)^−1^, or 600 mg·day^−1^; and placebo.

However, no significant results were observed in the comparison between the treated groups and the placebo group, and there was no reduction in the patients’ seizure rate. The same lack of significant results was found in trial NCT02072824, which involved 175 participants with an average age of 28.2 ± 12.6 years, divided into groups receiving 7 mg·(kg·day^−1^)^−1^ or 6 mg·(kg·day^−1^)^−1^; 14 mg·(kg·day^−1^)^−1^ or 12 mg·(kg·day^−1^)^−1^; and the placebo group. Nevertheless, among the analyses, no significant results were presented in when comparing the treated groups with the placebo group, and no reduction in the patients’ seizure rate was observed. The same results were obtained in trial NCT02072824, conducted with 175 participants, aged 28.2 ± 12.6 years, with the participants divided into groups receiving 6 mg·(kg·day^−1^)^−1^ or 7 mg·(kg·day^−1^)^−1^; 12 mg·(kg·day^−1^)^−1^ or 14 mg·(kg·day^−1^)^−1^; and placebo, in which no significant results were obtained when the experimental groups were compared to the placebo group.

### 5.13. Retigabine

Some drugs, such as retigabine, a drug that inhibits neurotransmission through the modulation of potassium channels [125], have shown efficacy in treating partial-onset seizures. The drug was used in trial NCT01777139, which involved 30 participants with a mean age of 36.0 ± 9.25 years. The participants underwent treatment with the drug and were evaluated, resulting in a 50% decrease in seizures over 28 days. Out of the 30 participants, 23 exhibited a positive response. However, some serious adverse events were reported, including headaches and diabetic retinopathy.

### 5.14. Padsenovil

New drugs, such as padsenovil (PSL), are still under study for the treatment of patients with seizures or epilepsy. Padsenovil’s mechanism of action is not fully elucidated, but it is known that it does not bind directly to the GABA receptors. However, it exhibits an antiepileptic action [126]. Trials involving this new drug were conducted, i.e., NCT02495844 and NCT03373383, which involved 55 and 411 participants, respectively. In study NCT03373383, the participants had a mean age of 39.8 ± 12.4 years. The trial revealed the efficacy of PSL compared to placebo, but without dose-dependent characteristics, while also demonstrating safety.

### 5.15. Other Clinical Trials

Some trials, such as NCT02721069 and NCT02724423, aim to evaluate the safety of certain drugs in new pharmaceutical forms. These trials divided participants into groups receiving different doses of NRL-1, an intranasal formulation of DZP, which is a benzodiazepine [127]. Another trial, NCT01999777, analyzed the efficacy and safety of intranasal midazolam, USL261 [128].

Trials NCT02926898 and NCT02682927, which involved 87 and 362 participants, respectively, aimed to evaluate the action of fenfluramine hydrochloride, a drug that increases the extracellular concentration of serotonin and acts as a 5-HT2 receptor agonist and a σ1 receptor antagonist. This mechanism allows for antiepileptic activity [129,130,131]. Both trials showed significant reductions in the frequency of monthly seizures compared to that observed in the placebo group, leading to increased quality of life, with seizure-free periods of up to several days [129,131].

Other trials using emerging molecules for the treatment of epilepsy aim to understand the pharmacokinetics, pharmacodynamics, and possible adverse effects of their use. Studies such as NCT03283371, NCT01866111, NCT03116828, and NCT02036853 continue to seek better seizure control or, in the case of NCT02564029, to focus on increasing the tolerability in cases of seizures caused by photosensitivity.

## 6. Non-Pharmacological Strategies for Epilepsy Treatment

### 6.1. Ketogenic Diet

The ketogenic diet is a type of diet based on the control of the proportion of lipid, protein, and carbohydrate intake. The main characteristic of the ketogenic diet (KD) is to mimic the fasting process, contributing to the process of producing ketone bodies [132]. KD intake is rich in fatty acids, low in carbohydrates, and adequate in protein supply; it has been used since 1920 to treat patients with epilepsy who do not respond well to drug treatments [132]. The mechanism of action of the diet has not yet been completely elucidated; however, it is understood that there is a relationship between mitochondrial function, the influence of ketone bodies on neural function, neurotransmitters modulating effects such as the increased synthesis of γ-aminobutyric acid, and the potential for membrane hyperpolarization. The mechanism of action of the ketogenic diet may also be associated with decreasing the release of glutamate, norepinephrine, adenosine, and fatty acids that contribute to the antiepileptic effects and stabilization of glycemia [133].

There are different approaches to applying the ketogenic diet, differing in food bases, such as the KD of long-chain triglycerides (LCT), the KD of medium-chain triglycerides (MCT), the modified Atkins diet (MAD), and treatment with low sugar levels [132]. The distribution of proportions and also the composition of the diets are both factors that individualize the treatment and contribute to its effectiveness and safety [134].

#### Types of Ketogenic Diets

LCT KD is a diet that is based on the proportion of lipid, protein, and carbohydrate intake, with the majority of the diet being lipids. In LCT KD, the patient’s hospitalization helps with adherence to treatment, and the lipid intake ratio is 4:1 (lipids: proteins + carbohydrates), which can range from 3:5:1 to 3:1 [135]. The MCT-type diet proposes the use of medium-chain lipids, such as octanoic and decanoic acid, lipids that are rapidly converted to ketone bodies by the liver [136].

MAD KD, unlike LCT, does not require the patient to be hospitalized, and 65% of the calories in the diet come from lipids. MAD KD allows caregivers, or even the patients themselves, greater flexibility in treatment, as well as better acceptability [135]. The low glycemic level diet allows for the consumption of carbohydrates; however, only those with a low glycemic level, which prevents a rapid increase in blood glucose levels [137]. 

The mechanism of action of ketogenic diets is based on the conversion of lipids into ketone bodies and their oxidation by the mitochondria, replacing glucose as an energy source for the brain [138]. The presence of ketone bodies and fatty acids is responsible for regulating the excitability of the plasma membrane. These mechanisms occur due to the influence of ketogenic diets in increasing the action of neurotransmitters such as γ-aminobutyric acid (GABA) [132]. Furthermore, under conditions of ketosis, there is a reduction in the use of the glycolytic pathway that produces adenosine triphosphate (ATP), a molecule that sensitizes the potassium channels, leading to cellular hyperpolarization, thus reducing the electrical excitability in seizures [132,138].

### 6.2. Neuromodulation Therapy

Neuromodulation therapy acts as a non-pharmacological treatment for epilepsy; this type of therapy directly stimulates or prevents the conduction of electrical potential in the brain [139]. Neuromodulation acts directly on the electrical conduction system of the CNS, modulating or modifying brain excitability and impacting the intensity and frequency of seizures in cases of epilepsy [140,141]. The methods used in treatment can be less invasive, through the dermal surface, or highly invasive, accessing the most cortical to the deepest regions of the brain [140].

Among the invasive treatments are: vagus nerve stimulation, deep brain stimulation, and responsive neural stimulation [142]. These are treatments that require implants in specific regions of the CNS, modulating the electrical stimuli that are responsible for triggering high activity in the hypersynchronized neural network during the seizure process [142]. While other treatments have the benefit of not requiring invasive procedures, these methods are used to stimulate the brain through waves, and even through sensorial methods. These treatments include transcranial magnetic stimulation (TMS), transcranial direct current stimulation (TDCS), ultrasound stimulation, transcutaneous VNS (UST-VNS), and trigeminal nerve stimulation (TNS) [142,143].

#### 6.2.1. Invasive

##### Vagus Nerve Stimulation

Vagus nerve stimulation (VNS) employs stimulus specifically to the vagus nerve, a capacity discovered when it was noted that massage and compression in the carotid region demonstrated the ability to suppress seizures [144], also taking into account the role of the vagus nerve in the control of the brainstem autonomic system [145]. The use of VNS has no age restrictions and can be applied to different types of seizures. The method consists of implanting an impulse generation system (implanted in the subcutaneous region of the left side of the chest), with wires connected to the vagus nerve (subcutaneous) connected to the commercially available programmable pulse generator device (NCP System; Cyberonics, Inc.; Houston, TX, USA) that controls all the parameters used to control seizures [144]. The pulse programming includes the following parameters: current load (the intensity of the electrical stimulus, in milliamps), the pulse width (duration of the electrical pulse, in microseconds), the pulse frequency, and the duty cycle (time to turn the stimulus on and off) [144,146,147].

##### Deep Brain Stimulation

Deep brain stimulation (DBS) is also an invasive neurointerventional technique, achieved using the implantation of an electrical stimulation compass and electrodes in specific locations in the brain [148]. Due to the characteristics of epilepsy in a hypersynchronized high-intensity discharge, DBS targets the regions of the anterior thalamic nucleus (ANT), the centromedian thalamic nucleus (CM), The subthalamic nucleus (SN), the caudate nucleus (CN), the cerebellum, and the hippocampus [149]. The mechanism of action of the intervention has not yet been completely elucidated; however, it is known that a high-frequency stimulus occurs in the implanted region, which cancels the stimulation of low frequency pathological synchronized neural activity [149], in addition to causing a rhythmic stimulus, helping to synchronize the thalamocortical region and preventing the disorganized stimulation of the cortical area, leading to seizures [150,151].

##### Responsive Neural Stimulation

This treatment is carried out through the implantation of equipment that maps the electrical activity of the brain, through electrocortigraphic activity and also sends stimuli to interrupt signs of seizure [152]. One to two electrodes are implanted in regions of the brain, according to the seizure focus. The electrodes’ function is to monitor brain activity and send electrical stimuli to prevent seizures [153]. Tools are used to detect seizure activity in real-time, as well as to monitor peaks and rhythmic activities within specific frequency bands, where the amplitude and duration of the “half-wave” records are analyzed. Additionally, a comparison is made between short-term averages (128 ms to 4 s) and a long-term average (4 s to 16 min) to identify changes in signal amplitude and frequency, along with the overall signal energy [153].

#### 6.2.2. Non-Invasive

##### Transcranial Magnetic Stimulation and Transcranial Direct Current Stimulation

As an alternative to surgery, these techniques some of the emerging treatments for epilepsy, as they are non-invasive and focal, with cortical and safe stimulation, emitting small intracranial electrical currents induced by strong and fluctuating extracranial fields [154,155,156]. There are different approaches to using TMS, which include single-pulse TMS, paired-pulse TMS, and repeated-pulse TMS [156].

TMS is a procedure that uses a coil to produce an electromagnetic field positioned on the head, in which the magnetic fields can modulate nerve cells, improving the symptoms of epilepsy [154]. This therapeutic intervention can reduce the hyperexcitability of nerve cells in the cortical region of the brain, producing a magnetic pulse (100–400μs), and causing depolarization in a few cm^2^ of the nearby axons [154].

tCDS, on the other hand, uses a weaker current (around 2 mA), but it also reaches the cortical region of the brain, promoting a change in the polarity of membrane potentials. This type of cathodic stimulation causes a decrease in the discharges linked to epileptic episodes [157]. tDCS can be used in cases of generalized and focal epilepsy, without age restrictions, and it is cheaper when compared to TMS [157].

##### Ultrasound Stimulation

Ultrasound stimulation occurs in the form of mechanical pressure exerted at a frequency >20 kHz. Neuromodulation using this method has demonstrated results in improving the nature of acute seizures [158]. As a mechanism related to the use of US, it is understood that the applied frequency can affect neural oscillations, which can desynchronize the rhythmic discharges responsible for seizure conditions and even affect the opening of the ion channels present in the neurons, causing cell hyperpolarization [159].

##### Transcutaneous VNS

As a non-invasive use of VNS, tVNS also stimulates the vagus nerve, with a mechanism similar to that of VNS, but which is applied to the vagus nerve of the auricular branch composed of three nerves: the vagus, glossopharyngeal, and facial nerves [160,161]. Transcutaneous VNS is a modern, non-invasive method which brings comfort and safety to the patient; the use of this approach has already demonstrated beneficial results, reducing seizures by 50% [162,163].

##### Trigeminal Nerve Stimulation

This method is also a non-invasive technique that helps in epilepsy treatment by anticipating tonic–clonic and generalized seizures [139]. The mechanism of TNS is a similar to that of VNS, where the trigeminal nerve is part of the nucleus of the solitary tract (NTS) and locus coeruleus (LC), brain regions that are involved in seizure reduction [139,140,157,164].

## 7. Risk Factors and Comorbidities Related to Epilepsy and AED Treatment

The World Health Organization reports that around 50% of epilepsy cases worldwide remain unknown, and of the 50% which are known, it is estimated that 25% are preventable cases, and 70% are diagnoses in which the crisis can be eliminate with the use of medication [165]. Epilepsy can also trigger comorbidities, just as the disease itself can be triggered by other pathologies and their associated comorbidities. According to the World Health Organization, it is also possible to divide the causes of epilepsy into categories including structural, genetic, infectious, metabolic, immune, and unknown [165].

As a neurological disease, epilepsy brings with it the burden of various adjacent comorbidities. Comorbidities can be defined as the combination of two or more diseases that work together. Epilepsy, as a disease of great social impact, can be accompanied by depression, which directly interferes with the form and quality of life of the affected individual, as it can also cause neurological disorders, given its action on the nervous system [166]. Studies show that about 50% of adults with epilepsy exhibit at least one comorbidity, and that one in three people express psychiatric comorbidities, the most common of which are dementia and intellectual disability [166,167].

Recent studies have linked the higher rate of epilepsy in elderly people with two main factors, possibly related to its high incidence: first, the increased life expectancy of people who developed the disorder at a young age; and second, the fact that elderly people are more likely to develop cerebrovascular trauma, such as strokes, and neurodegenerative diseases, including Alzheimer’s disease. Some studies indicate that elderly people who exhibit these pathologies have an increased risk of developing epilepsy later in life [168].

It was also observed that elderly people with epilepsy had more cognitive problems than elderly people without the condition, showing that this disorder can influence the quality of life of these individuals [168]. In addition to the elderly, epilepsy in children has been widely reported. Along with this, affected individuals tend to develop other comorbidities, as observed in other age groups. Migraines, depression, anxiety, attention deficit, sleep disorders, and autism spectrum disorder are among the main problems that affect children with epilepsy [169].

Neuropsychiatric comorbidities are those that stand out most when it comes to epilepsy, mainly because epilepsy is a neurological disease. Therefore, it is important to highlight the epilepsy–dementia and dementia–epilepsy relationship. A meta-analysis study reported that late-onset epilepsy in elderly people is related to a higher risk of developing Alzheimer’s disease and vascular dementia [170].

Another risk factor for the onset of epilepsy is the occurrence of a stroke. A study in Taiwan recorded the development of epilepsy in 402 young people after ischemic stroke from a cohort of 6512 patients. The unhealthy behavior of patients was one of the factors noted for the development of ischemic stroke, with a 2.90-fold greater instance of drug abuse in these patients, whereas the use of statins decreases the risk of developing stroke [171].

It was observed that people with Alzheimer’s disease were more likely to experience seizures and develop epilepsy when compared to those who did not have this neurodegenerative condition [172]. In addition to the risk factors discussed here and the genetic influences that will be discussed later, there is also a relationship between the appearance of cognitive impairment and the drug treatment prescribed to the patient. Various drugs present several indications of adverse reactions on the neurological system, with some studies even indicating an increased risk of developing neurodegenerative diseases, including different forms of dementia (Table 2).

Some studies have shown the relationship between some medications aimed at treating epilepsy and the appearance of symptoms that can predict the development of dementia. Among these, we highlight topiramate and zonisamide, for which, among the common adverse effects, changes in memory have been reported [173].

Valproic acid, for example, is a medication that, despite dementia not being noted as one of the main adverse effects, presents case reports indicating a possible relationship between the chemical and the appearance of different types of dementia in patients [174]. Another study analyzing the relationship of valproic acid in the treatment of bipolar disorder also showed that the risk of developing dementia was increased in around 73–95% of patients who used this medication [175].

Studies have shown that drugs from the benzodiazepine class, including clonazepam, diazepam, clobazam, and lorazepam, are among the main drugs that depress cognitive activity when used in high dosages [176]. Other studies were carried out to observe the effectiveness and tolerability of drugs used to treat Alzheimer’s patients suffering from epilepsy; it was observed that among the drugs tested, lamotrigine and phenobarbital seemed to worsen the patients’ cognition [172].

One study noted that the incidence of anxiety and depression was increased in children and adolescents with epilepsy by about 18.9 and 13.5%, respectively, when compared to the levels in healthy children. The same was observed concerning attention deficit hyperactivity disorder (ADHD), with an increased incidence between 2.5- and 5.5-fold more in children and adolescents with epilepsy [177]. However, the relationship between ADHD and epilepsy is not well established, but some studies suggest that the medication used in the treatment of epilepsy, such as phenobarbital, for example, can induce the onset of symptoms related to ADHD [178].

Concerning depression, many studies have shown that there is a relationship between the increased development of epilepsy in people with depression, suggesting similar mechanisms between the two diseases, such as altered serotonin levels and/or the common regions of the brain that are affected by both pathologies. These investigations show that the relationships between the two diseases can occur bidirectionally [169,179]. The emergence of depression due to epilepsy or epilepsy due to febrile seizures are examples of coexisting comorbidities. Epilepsy may favor the development of depression through exposure to chronic stress. Increased interleukin-1β (IL-1β) signaling in the hippocampus may be a factor for temporal lobe epilepsy (TLE), as well as for clinical depression; however, glutamate has been suggested as a potential pathogen for depression [180].

Some studies relate the appearance of depression and anxiety in patients with epilepsy to the medications used, such as levetiracetam, which has been shown to have a significant influence on increasing the rate of depression and irritability in epileptic patients. In addition to this, zonisamide has also been shown to have an important influence on the appearance of depressive conditions [181].

Despite the relationship between febrile seizures and epilepsy, neurodevelopmental delay and electroencephalogram abnormalities in children are major risk factors for the development of epilepsy and febrile seizures. Long-term prophylaxis treatments do not decrease recurrence [182]. Febrile seizures and electroencephalogrammay also be risk factors for drug-resistant epilepsy, as well as status epilepticus, symptomatic etiology, and other types of seizures. Neurodevelopmental delay, poor outcome of short-term therapy, and high initial seizure frequency are not risk factors for drug-resistant epilepsy [183].

Depression as a comorbidity related to epilepsy also raises an alert for the increase in suicide cases among those affected, arousing the interest of the scientific community in the diagnosis and treatment of this comorbidity to reduce the incidence of suicide cases [179]. The increased incidence of suicide or suicide attempts may also be related to drug treatments, such as the use of phenobarbital and pregabalin [181].

In addition to these diseases, epilepsy also shows a strong connection with autism spectrum disorders (ASD), and studies demonstrated that the simultaneous occurrence of the two pathologies may be associated with genetic factors or external agents directly related to pregnancy, such as acquired diseases [184]. A systematic study pointed out that about 12% of autistic individuals had been diagnosed with epilepsy, and that 9% of individuals with epilepsy also had ASD [184]. Another study showed that the incidence of epilepsy in individuals with ASD in adulthood was higher than in childhood, and that the occurrence was more prevalent in female individuals [185]. 

Some studies have suggested that there are mechanisms in common between epilepsy and ASD, such as the hyperactivation of mTOR signaling through genetic mutations; this signaling pathway is related to the control of cell growth and was discovered based on rapamycin, which works as an inhibitor of this pathway and is used in the control of some pathologies, such as rheumatoid arthritis and atherosclerosis. A study with animal models showed that rapamycin exhibited positive results in individuals with ASD and epilepsy [186,187,188,189]. In addition to this, another relationship was observed between ASD and epilepsy, i.e., the absence or low signaling of the GABAergic marker, which causes an imbalance in the cerebral cortex of humans, as well as in animal models [187].

Furthermore, a study sought to analyze the influence of antiepileptic medicines on children who were exposed to these chemicals during pregnancy. It had been reported that some medications, such as oxcarbazepine, valproate, lamotrigine, and lamotrigine concomitantly valproate, increased the risk of developing ASD in those involved in the study [190].

In this way, we verified that the appearance of comorbidities related to epilepsy may be directly related to the implications of the disease itself, as well as be triggered by the use of prescribed medications. Other non-neuropsychiatric side effects of AEDs include hypersensitivity, serious dermatologic reaction, hepatotoxicity, and withdrawal (Table 2). This demonstrates the need for the characterization of the mechanisms of action of current commercially available AEDs and the development of new AEDs with fewer side effects and less impact on comorbidities [191,192,193].

## 8. Conclusions

Epilepsy is a complex symptomatic disease with several risk factors, often associated with a strong genetic predisposition, rather than a single expression and cause. Advances in genomic technology have revealed the complex genetic architecture behind epilepsies. In recent years, we have seen the elucidation of several therapeutic targets aimed at the development of AEDs. To be considered an anticonvulsant, a drug must balance the excitation and inhibition of the neurotransmitters, especially in the GABA and glutamate pathways. The main targets of the mechanisms of action are the molecular agents that act directly on the physiological processes of neuronal actions, including ion channels, enzymes, transport proteins, receptors, and regulators of gene expression. These agents allow for the partial or total reduction of the symptoms observed in epilepsy. The mechanisms of action of most commercially available AEDs are proposed to target, individually or simultaneously, the GABA system, the voltage-gated channels, the synaptic vesicle protein 2A or the α-amino-3-hydroxy-5-methyl-4-isoxazolepropionic acid (AMPA) glutamate receptor, or the N-methyl-D-aspartate (NMDA) receptor.

Epilepsy represents a great burden in terms of quality of life, morbidity, and risk of premature mortality, especially for those who continue to have seizures despite treatment. In addition, comorbidities have been increasingly recognized as important etiologic and prognostic markers. Although anticonvulsant drugs can suppress seizures in up to two-thirds of patients, they fail to alter the long-term prognosis and can be associated with numerous side effects, as well has have an impact on comorbidities. Despite being the most effective way to achieve long-term seizure freedom, epilepsy surgery is only a viable alternative for patients with drug-resistant epilepsy. Non-pharmacological approaches can also be deployed concomitantly with drug intervention; these include a ketogenic diet and invasive and non-invasive neuromodulation therapies. In summary, the epilepsy etiology and pathophysiology must be better understood in order to elucidate known molecular targets and unravel novel targets to develop new forms of treatment with fewer side effects and less impact on comorbidities.

## Figures and Tables

**Table 1:** Most relevant completed epilepsy-related clinical trials since 2013 yielding results involving drug intervention, with data collected from ClinicalTrials.gov. NCT refers to National Clinical Trial; M and F refer to male and female.

NCT Number	Conditions	Interventions	Sex	Age	Phase
NCT02451696	Epilepsy | Tuberous Sclerosis Complex | Focal Cortical Dysplasia	Everolimus	M/F	Child, adult	2
NCT02758626	Epilepsy	Ataluren | Placebo	M/F	Child	2
NCT03940326	Epilepsy, Idiopathic Generalized	Levetiracetam | Valproate	M/F	Child, adult	4
NCT02564952	Epilepsy	GWP42003-P | Clobazam	M/F	Adult	2
NCT03650452	Epilepsy | Dravet Syndrome | Lennox–Gastaut Syndrome	Tak-935 | Placebo	M/F	Child	2
NCT01777139	Epilepsy	Retigabine Immediate Release	M/F	Adult	3
NCT03179891	Epilepsy	Diazepam Buccal Film	M/F	Adult	2
NCT03283371	Epilepsy, Focal Seizures, Partial Seizures	Natalizumab | Placebo	M/F	Adult	2
NCT01963208	Drug Resistant Partial Onset Seizure	Ganaxolone | Placebo	M/F	Adult	3
NCT03478982	Epilepsy	Staccato alprazolam | Placebo	M/F	Adult	2
NCT03222349	Epilepsy	Diazepam Buccal Film	M/F	Child	2
NCT02682927	Dravet Syndrome | Seizure Disorder	Zx008 (fenfluramine hydrochloride) | Placebo	M/F	Child, adult	3
NCT02224703	Epilepsy | Dravet Syndrome	GWP42003-P | Placebo	M/F	Child, adult	3
NCT02565108	Epilepsy	GWP42003-P | Clobazam	M/F	Adult	2
NCT02849626	Partial-Onset or Primary Generalized Tonic–Clonic Seizures	Perampanel	M/F	Child	3
NCT02700412	Epilepsy | Seizures	Epidiolex	M/F	Adult	1
NCT02724423	Acute Repetitive Seizures	Nrl-1	M/F	Child, adult	1
NCT02695537	Epilepsy | Seizures	Epidiolex	M/F	Child, adult	1
NCT03405714	Epilepsy	Brivaracetam	M/F	Child	2
NCT03373383	Drug-Resistant Epilepsy | Focal-Onset Seizures	Padsevonil | Placebo	M/F	Adult	2
NCT01747915	Generalized Tonic–Clonic Seizures	Pregabalin | Placebo	M/F	Child, adult	3
NCT02404168	Epilepsy	Lamotrigine (Brand Lamictal) | Lamotrigine (Generic Teva)	M/F	Adult	4
NCT02721069	Acute Repetitive Seizures | Breakthrough Seizures	NRL-1	M/F	Child, adult	3
NCT01954121	Epilepsy | Partial Seizures	Levetiracetam | Carbamazepine	M/F	Child, adult	3
NCT01832038	Epilepsy | Partial-Onset Seizures	Lacosamide	M/F	Child, adult	3
NCT03428360	Epilepsy	Diazepam Buccal Soluble Film	M/F	Child, adult	3
NCT02036853	Glucose Transporter Type-1 Deficiency Syndrome (Glut1 DS)	Triheptanoin	M/F	Child, adult	2
NCT01964560	Epilepsy	Lacosamide	M/F	Child	3
NCT02224560	Epilepsy | Lennox–Gastaut Syndrome	GWP42003-P | Placebo	M/F	Child, adult	3
NCT02224573	Epilepsy | Dravet Syndrome | Lennox–Gastaut Syndrome	GWP42003-P	M/F	Child, adult	3
NCT03021018	Epilepsy	Brivaracetam | Lorazepam	M/F	Adult	2
NCT01999777	Epilepsy	USL261 | Placebo	M/F	Child, adult	3
NCT01713946	Tuberous Sclerosis Complex-Associated Refractory Seizures	RAD001 | Placebo | Antiepileptic drug (1 to 3 only) | Open Label RAD001	M/F	Child, adult	3
NCT02564029	Reflex Epilepsy, Photosensitive	PF-06372865 | Placebo | Lorazepam	M/F	Adult	2
NCT03116828	Epilepsy with Partial Onset Seizures	Eslicarbazepine Acetate (first add-on) | Eslicarbazepine Acetate (late add-on)	M/F	Adult	4
NCT02100644	Epilepsy	Lamotrigine Tablets	F	Child, adult	4
NCT02495844	Highly Drug-Resistant Focal Epilepsy	UCB0942 | Placebo	M/F	Adult	2
NCT01866111	Partial Epilepsy	Ykp3089 | Placebo	M/F	Adult	2
NCT02926898	Dravet Syndrome	Zx008 (fenfluramine hydrochloride) | Matching Placebo	M/F	Child, adult	3
NCT02351115	Epilepsy	Placebo | Inhaled Alprazolam | Inhaled Alprazolam | Inhaled Alprazolam | Placebo	M/F	Adult	2
NCT03953820	Epilepsy	Diazepam Buccal Film | Diastat^®^ Rectal Gel	M/F	Adult	1|2
NCT02072824	Partial Onset Seizures	Pregabalin dose level 1 | Pregabalin dose level 2 | Placebo	M/F	Child	3
NCT04882540	Healthy Participants	Brivaracetam	M/F	Adult	1
NCT02726074	Epilepsy	Perampanel	M/F	Child, adult	4

**Table 2:** Side effects of FDA-approved antiepileptic drugs (AEDs); data were collected from the Epilepsy Foundation (https://www.epilepsy.com/ (accessed on 1 October 2023)) and DailyMed (https://dailymed.nlm.nih.gov/dailymed/ (accessed on 1 October 2023)).

Side Effects	Drug	
Suicidal behavior and ideation	Brivaracetam	Lamotrigine
	Cannabidiol Oral Solution	Levetiracetam-XR
	Carbamazepine	Midazolam Nasal
	Carbamazepine-XR	Oxcarbazepine
	Cenobamate	Perampanel
	Clobazam	Phenytoin
	Clonazepam	Pregabalin
	Diazepam Nasal	Primidone
	Divalproex Sodium	Rufinamide
	Eslicarbazepine Acetate	Stiripentol
	Ethosuximide	Topiramate
	Felbamate	Topiramate XR
	Fenfluramine	Valproic Acid
	Gabapentin	Fenfuramine
	Lacosamide	Zonisamide
Psychiatric adverse reaction	Brivaracetam	Perampanel
	Fenfluramine	Phenobarbital
	Gabapentin	Pregabalin
	Levetiracetam	Tiagabine Hydrochloride
	Levetiracetam-XR	Topiramate
	Lorazepam	Topiramate XR
	Midazolam Nasal	Zonisamide
	Oxcarbazepine	
Neurological Adverse Reactions	Brivaracetam	Lorazepam
	Cannabidiol Oral Solution	Midazolam Nasal
	Cenobamate	Oxcarbazepine
	Clobazam	Perampanel
	Clonazepam	Phenobarbital
	Diazepam Nasal	Pregabalin
	Diazepam Rectal	Primidone
	Divalproex Sodium	Rufinamide
	Divalproex Sodium-ER	Stiripentol
	Eslicarbazepine Acetate	Tiagabine Hydrochloride
	Felbamate	Topiramate
	Fenfluramine	Topiramate XR
	Gabapentin	Valproic Acid
	Lacosamide	Fenfuramine
	Levetiracetam	Zonisamide
	Levetiracetam-XR	
Hypersensitivity	Brivaracetam	Lacosamide
	Cannabidiol oral solution	Lamotrigine
	Carbamazepine	Oxcarbazepine
	Carbamazepine-XR	Phenobarbital
	Cenobamate	Phenytoin
	Eslicarbazepine Acetate	Pregabalin
	Gabapentin	
Withdrawal of AEDs	Brivaracetam	Lorazepam
	Cannabidiol oral solution	Midazolam Nasal
	Cenobamate	Oxcarbazepine
	Clobazam	Perampanel
	Clonazepam	Phenobarbital
	Diazepam Nasal	Phenytoin
	Diazepam Rectal	Rufinamide
	Eslicarbazepine Acetate	Stiripentol
	Fenfluramine	Tiagabine Hydrochloride
	Gabapentin	Topiramate
	Lacosamide	Topiramate XR
	Lamotrigine	Fenfuramine
	Levetiracetam	Zonisamide
	Levetiracetam-XR	
Hepatotoxicity	Cannabidiol Oral Solution	Phenytoin
	Divalproex Sodium	Valproic Acid
	Divalproex Sodium-ER	
Serious Dermatologic Reactions	Carbamazepine	Levetiracetam-XR
	Carbamazepine-XR	Oxcarbazepine
	Clobazam	Phenobarbital
	Eslicarbazepine Acetate	Phenytoin
	Ethosuximide	Topiramate
	Lamotrigine	Zonisamide
	Levetiracetam	

## Data Availability

Publicly available datasets were analyzed in this study. These data can be found at https://clinicaltrials.gov/ (accessed on 10 July 2023).

## Supplementary Materials

The following supporting information can be downloaded at: https://www.mdpi.com/article/10.3390/brainsci14010071/s1. Supplementary Table S1. FDA-approved drug used for the treatment of seizures, data collected from the Epilepsy Foundation (https://www.epilepsy.com/) and DailyMed (https://dailymed.nlm.nih.gov/dailymed/).
