# Supplementary material for: Pathophysiology to Risk Factor and Therapeutics to Treatment Strategies on Epilepsy
Source: Brain Sci. 2024 Jan 10;14(1):71. doi: 10.3390/brainsci14010071 (PMC10813806; doi:10.3390/brainsci14010071)
Supplement: Supplementary file 1 [file brainsci-14-00071-s001.zip › brainsci-2806186-supplementary.pdf]

Supplementary Table S1. FDA-approved drug used for the treatment of seizures, data collected from the Epilepsy Foundation (https://www.epilepsy.com/) and DailyMed (https://dailymed.nlm.nih.gov/dailymed/).

Drug	Commercial name ®	Mechanism of action	Initial daily dose	Daily dose	Used for	Most common side effects
**Brivaracetam**	Briviact	Unknown, however it displays a high and selective affinity for synaptic vesicle protein 2A (SV2A) in the brain, which may contribute to the anticonvulsant effect	50 mg	100 to 200 mg	Focal (partial) seizures	Dizziness<br>Feeling tired or sleepy<br>Nausea and vomiting
**Cannabidiol oral solution**	Epidiolex	Unknown, however it may change calcium levels in brain cells that are important for sending messages or signals between cells. The medicine may also decrease inflammation in the brain	5 mg:per:kg	10 to 20 mg:per:kg	Seizures associated with Lennox-Gastaut syndrome, Dravet syndrome, or tuberous sclerosis complex in patients 1 year of age and older	Sleepiness<br>Decreased appetite<br>Diarrhea<br>Increase in liver enzymes<br>Rash<br>Sleep problems<br>Infections
**Carbamazepine**	Tegretol, Epitol	Slow inhibition of voltage-gated sodium channels in the brain cells, preventing repetitive and sustained firing of an action potential	100 to 200 mg	600 to 1200 mg	Focal or partial seizures<br>Generalized tonic-clonic seizures<br>Mixed seizure types (both generalized and focal or partial)	Blurry or double vision<br>Dizziness<br>Headache<br>Nausea or upset stomach<br>Sleepiness or feeling tired<br>Unsteady walking

**Carbamazepine-XR**	Carbatrol, Tegretol XR	Unknown, however it appears to act by reducing polysynaptic responses and blocking the post-tetanic potentiation	100 to 200 mg	600 to 1200 mg	Focal Impaired Awareness or Complex Partial Seizures	Nausea<br>Vomiting<br>Dizziness<br>Drowsiness<br>Constipation<br>Dry mouth<br>Unsteadiness
**Cenobamate**	Xcopri	Unknown, however can reduce repetitive neuronal firing by inhibiting voltage-gated sodium currents. It is also a positive allosteric modulator of the γ-aminobutyric acid (GABAA) ion channel	12.5 mg	200 to 400 mg	Focal Aware Onset Seizure	Feeling sleepy and tired<br>Dizziness<br>Double Vision<br>Headache
**Clobazam**	Sympazan, Onfi	Unknown but is thought to involve potentiation of GABAergic neurotransmission resulting from binding at the benzodiazepine site of the GABA A receptor	5 to 10 mg	20 to 40 mg	Lennox-Gastaut Syndrome<br>Absence Seizures<br>Atonic Seizures<br>Atypical Absence Seizures<br>Focal Impaired Awareness or Complex Partial Seizures<br>Myoclonic Seizures<br>Refractory Seizures<br>Secondarily Generalized Seizures or Bilateral Tonic Clonic Seizure<br>Focal Aware Onset Seizure<br>Tonic-clonic Seizures	Drowsiness<br>Dizziness<br>Poor coordination<br>Drooling<br>Restlessness or aggressiveness
**Clonazepam**	Klonopin	Unknown, although it is believed to be related to its ability to enhance the activity of gamma aminobutyric acid (GABA), the major inhibitory neurotransmitter in the central nervous system	1.5 mg	≤20 mg	Lennox-Gastaut Syndrome<br>Absence Seizures<br>Myoclonic Seizures	Tiredness<br>Dizziness<br>Unsteadiness<br>Impaired attention and memory<br>Irritability<br>Hyperactivity

						Drooling<br>Depression<br>Nausea<br>Loss of appetite
**Diazepam Nasal**	Valtoco	Unknown but it is thought to involve potentiation of GABAergic neurotransmission resulting from binding at the benzodiazepine site of the $GABA_A$ receptor	5 to 20 mg	5 to 20 mg	Absence Seizures<br>Atonic Seizures<br>Atypical Absence Seizures<br>Clonic Seizures<br>Focal Impaired Awareness or Complex Partial Seizures<br>Febrile Seizures<br>Myoclonic Seizures<br>Refractory Seizures<br>Secondarily Generalized Seizures or Bilateral Tonic Clonic Seizure<br>Focal Aware Onset Seizure<br>Tonic Seizures<br>Tonic-clonic Seizures<br>Unknown Onset	Dizziness<br>Headache<br>Pain<br>Nervousness<br>Diarrhea<br>Unsteady walking<br>Behavior change<br>Poor coordination<br>Asthma<br>Runny nose<br>Rash (2 to 5 %)<br>Vasodilation
**Diazepam Rectal**	Diastat	Suppress seizures through an interaction with γ-aminobutyric acid (GABA) receptors of the A-type (GABA A). GABA, the major inhibitory neurotransmitter in the central nervous system (CNS), acts at this receptor to open the membrane channel allowing chloride ions to flow into neurons	5 to 20 mg	5 to 20 mg	Lennox-Gastaut Syndrome<br>Absence Seizures<br>Atonic Seizures<br>Focal Impaired Awareness or Complex Partial Seizures<br>Myoclonic Seizures<br>Focal Aware Onset Seizure<br>Tonic-clonic Seizures	Tired or sleepy<br>Poor coordination<br>Behavior changes<br>Blurred or double-vision<br>Dizziness<br>Headache
**Divalproex Sodium**	Depakote, Depacon	Divalproex sodium dissociates to the valproate ion in the gastrointestinal tract. The mechanisms by which valproate exerts its therapeutic effects have not been	10 to 15 $mg.kg^{-1}$	≤60 $mg.kg^{-1}$	Absence Seizures<br>Focal Aware Onset Seizure	Tiredness<br>Dizziness<br>Upset stomach<br>Vomiting

		established. It has been suggested that its activity in epilepsy is related to increased brain concentrations of gamma-aminobutyric acid (GABA)				Tremor<br>Hair loss<br>Weight gain<br>Changes in behavior
**Divalproex Sodium-ER**	Depakote ER	Divalproex sodium dissociates to the valproate ion in the gastrointestinal tract. The mechanisms by which valproate exerts its therapeutic effects have not been established. It has been suggested that its activity in epilepsy is related to increased brain concentrations of gamma-aminobutyric acid (GABA)	10 to 15 $mg.kg^{-1}$	≤60 $mg.kg^{-1}$	Absence Seizures<br>Focal Aware Onset Seizure	Tiredness<br>Dizziness<br>Upset stomach<br>Vomiting<br>Tremor<br>Hair loss<br>Weight gain<br>Changes in behavior
**Eslicarbazepine Acetate**	Aptiom	Unknown but is thought to involve inhibition of voltage-gated sodium channels	400 mg	800 to 1600 mg	Temporal Lobe Epilepsy<br>Focal Impaired Awareness or Complex Partial Seizures<br>Secondarily Generalized Seizures or Bilateral Tonic Clonic Seizure<br>Focal Aware Onset Seizure	Clumsy, problems with coordination<br>Dizziness<br>Double vision or blurry vision<br>Headache<br>Nause or upset stomach<br>Problems with thinking, memory or attention (cognitive problems)<br>Sleepiness<br>Tremors of feeling shakey

						Vomiting
**Ethosuximide**	Zarontin	Ethosuximide suppresses the paroxysmal three cycle per second spike and wave activity associated with lapses of consciousness which is common in absence (petit mal) seizures. The frequency of epileptiform attacks is reduced, apparently by depression of the motor cortex and elevation of the threshold of the central nervous system to convulsive stimuli	250 to 500 mg	20 $mg.kg^{-1}$	Childhood and Juvenile Absence Epilepsy<br>Absence Seizures<br>Atypical Absence Seizures	Drowsiness<br>Dizziness<br>Tiredness<br>Headache<br>Stomach upset<br>Loss of appetite<br>Nausea<br>Vomiting<br>Weight loss<br>Diarrhea<br>Loss of coordination
**Felbamate**	Felbatol	Unknown, however it interacts as an antagonist at the strychnine-insensitive glycine recognition site of the NMDA receptor-ionophore complex, also has weak inhibitory effects on GABA-receptor binding	1200 mg	3600 mg	Lennox-Gastaut Syndrome<br>Temporal Lobe Epilepsy<br>Atonic Seizures<br>Focal Impaired Awareness or Complex Partial Seizures<br>Refractory Seizures<br>Secondarily Generalized Seizures or Bilateral Tonic Clonic Seizure<br>Focal Aware Onset Seizure<br>Tonic-clonic Seizures	Decreased appetite and weight loss<br>Nausea<br>Insomnia<br>Headache<br>Poor coordination or tremor<br>Vision problems<br>Dizziness<br>Vomiting<br>Mood changes or anxiety
**Fenfluramine**	Fintepla	Unknown	0.1 $mg.kg^{-1}$	0.35 $mg.kg^{-1}$	Seizures associated with Dravet syndrome and Lennox-Gastaut syndrome	Decreased appetite<br>Diarrhea<br>Weight loss

						Feeling tired or sleepy
**Gabapentin**	Neurontin	Unknown, it binds with high-affinity to the $\alpha 2\delta$ subunit of voltage-activated calcium channels; however, the relationship of this binding to the therapeutic effects of gabapentin is unknown	300 mg	900 mg	Focal Impaired Awareness or Complex Partial Seizures<br>Focal Aware Onset Seizure	Tiredness<br>Sleepiness<br>Dizziness<br>Weight gain<br>Ankle swelling
**Lacosamide**	Vimpat	Unknown, however *in vitro* it selectively enhances slow inactivation of voltage-gated sodium channels, resulting in stabilization of hyperexcitable neuronal membranes and inhibition of repetitive neuronal firing	200 mg	300 to 400 mg	Partial-Onset Seizures<br>Primary Generalized Tonic-Clonic Seizures	Coordination problems<br>Dizziness<br>Double vision<br>Headache<br>Nausea, vomiting<br>Sleepiness<br>Unsteady walking
**Lamotrigine**	Lamictal	Unknown, however *in vitro* pharmacological studies suggest that lamotrigine inhibits voltage-sensitive sodium channels, thereby stabilizing neuronal membranes and consequently modulating presynaptic transmitter release of excitatory amino acids	25 mg	225 to 375 mg	Partial-onset seizures<br>Primary generalized tonic-clonic seizures<br>Generalized seizures of Lennox-Gastaut syndrome	Dizziness<br>Upset stomach<br>Headache<br>Unsteadiness<br>Double vision<br>Rash
**Levetiracetam**	Keppra, Spritam	Unknown, however it is proposed that the interaction of levetiracetam with the SV2A protein may contribute to the antiepileptic mechanism of action of the drug	1000 mg	≤3000 mg	Partial-Onset Seizures<br>Myoclonic Seizures in Patients with Juvenile Myoclonic Epilepsy<br>Primary Generalized Tonic-Clonic Seizures	Dizziness<br>Headache<br>Irritability<br>Loss of strength and energy<br>Mood and behavior changes

						Sleepiness
**Levetiracetam XR**	Keppra XR	Unknown, however it is proposed that the interaction of levetiracetam with the SV2A protein may contribute to the antiepileptic mechanism of action of the drug	1000 mg	≤3000 mg	Focal Impaired Awareness or Complex Partial Seizures<br>Focal Aware Onset Seizure	Sleepiness<br>Loss of strength and energy<br>Dizziness<br>Anxiety<br>Nervousness<br>Irritability<br>Depression<br>Other mood changes
**Lorazepam**	Ativan	Interacts with the γ-aminobutyric acid (GABA)-benzodiazepine receptor complex	2 mg	4 mg	Benign Rolandic Epilepsy<br>Childhood and Juvenile Absence Epilepsy<br>Dravet Syndrome<br>Hypothalamic Hamartoma<br>Infantile Spasms/West's Syndrome<br>Juvenile Myoclonic Epilepsy<br>Lennox-Gastaut Syndrome<br>Rasmussen's Syndrome<br>Ring Chromosome 20 Syndrome<br>Reflex Epilepsies<br>Temporal Lobe Epilepsy<br>Absence Seizures<br>Atonic Seizures<br>Atypical Absence Seizures<br>Clonic Seizures<br>Focal Impaired Awareness or Complex Partial Seizures<br>Febrile Seizures<br>Myoclonic Seizures<br>Refractory Seizures	Poor coordination<br>Behavior changes<br>Unsteadiness, sleepiness<br>Fatigue<br>Drowsiness<br>Blurred or double-vision<br>Dizziness

					Secondarily Generalized Seizures or Bilateral Tonic Clonic Seizure<br>Focal Aware Onset Seizure<br>Tonic Seizures<br>Tonic-clonic Seizures<br>Developmental/Epileptic Encephalopathy with Spike Wave Activation In Sleep DEE-SWAS	
**Midazolam Nasal**	Nayzilam	Unknown, but it is thought to involve potentiation of GABAergic neurotransmission resulting from binding at the benzodiazepine site of the GABAA receptor	5 mg	10 mg	Absence Seizures<br>Atonic Seizures<br>Atypical Absence Seizures<br>Clonic Seizures<br>Focal Impaired Awareness or Complex Partial Seizures<br>Febrile Seizures<br>Myoclonic Seizures<br>Secondarily Generalized Seizures or Bilateral Tonic Clonic Seizure<br>Focal Aware Onset Seizure<br>Tonic Seizures<br>Tonic-clonic Seizures<br>Unknown Onset	Sleepiness<br>Headache<br>Runny nose<br>Nasal discomfort<br>Throat irritation
**Oxcarbazepine**	Trileptal, Oxtellar XR	Unknown; however, *in vitro* electrophysiological studies indicate that they produce blockade of voltage-sensitive sodium channels, resulting in stabilization of hyperexcited neural membranes, inhibition of repetitive neuronal firing, and diminution of propagation of synaptic impulses	600 mg	1200 to 2400 mg	Temporal Lobe Epilepsy<br>Focal Impaired Awareness or Complex Partial Seizures<br>Secondarily Generalized Seizures or Bilateral Tonic Clonic Seizure<br>Focal Aware Onset Seizure<br>Tonic-clonic Seizures	Dizziness<br>Double vision<br>Headache<br>Low sodium or salt levels in the bloodstream<br>Nausea, upset stomach, indigestion<br>Rash

						Tired, sleepy, or drowsy<br>Trembling of hands<br>Trouble walking<br>Trouble with coordination<br>Vomiting
**Perampanel**	Fycompa	Non-competitive antagonist of the ionotropic α-amino-3-hydroxy-5-methyl-4-isoxazolepropionic acid (AMPA) glutamate receptor on post-synaptic neurons	2 mg	8 to 12 mg	Temporal Lobe Epilepsy<br>Focal Impaired Awareness or Complex Partial Seizures<br>Secondarily Generalized Seizures or Bilateral Tonic Clonic Seizure<br>Focal Aware Onset Seizure<br>Tonic-clonic Seizures	Anxiety<br>Dizziness<br>Falls<br>Headache<br>Irritability<br>Problems with coordination<br>Sleepiness or tired<br>Stomach upset, nausea, vomiting<br>Unsteady walking or problems with balance<br>Vertigo<br>Weight gain
**Phenobarbital**	Luminal	Acts on GABAA receptors, increasing synaptic inhibition. This has the effect of elevating seizure threshold and reducing the spread of seizure activity from a seizure focus. Phenobarbital may also	60 to 200 mg	60 to 200 mg	Lennox-Gastaut Syndrome<br>Rasmussen's Syndrome<br>Temporal Lobe Epilepsy<br>Clonic Seizures<br>Focal Impaired Awareness or Complex Partial Seizures	Depression<br>Hyperactivity<br>Trouble paying attention<br>Dizziness

		inhibit calcium channels, resulting in a decrease in excitatory transmitter release			Refractory Seizures<br>Secondarily Generalized Seizures or Bilateral Tonic Clonic Seizure<br>Focal Aware Onset Seizure<br>Tonic Seizures<br>Tonic-clonic Seizures	Memory problems<br>Decreased sexual interest<br>Inability to have an erection<br>Slurred speech<br>Upset stomach<br>Anemia<br>Deficiency of the vitamin folic acid<br>Rash<br>Fever<br>Low calcium levels, bone loss
**Phenytoin**	Dilantin	Unknown, however is thought to involve the voltage-dependent blockade of membrane sodium channels resulting in a reduction in sustained high-frequency neuronal discharges	100 mg	300 mg	Temporal Lobe Epilepsy<br>Focal Impaired Awareness or Complex Partial Seizures<br>Refractory Seizures<br>Secondarily Generalized Seizures or Bilateral Tonic Clonic Seizure<br>Focal Aware Onset Seizure<br>Tonic-clonic Seizures	Jerking movements of the eyes<br>Decreased coordination<br>Shaking of the hands<br>Slowed thinking and movement<br>Memory problems<br>Slurred speech<br>Poor concentration
**Pregabalin**	Lyrica	Not fully elucidated, however it binds with high affinity to the alpha$_2$-delta site	150 mg	≤600 mg	Focal Impaired Awareness or Complex Partial Seizures	Dizziness<br>Imbalance

		(an auxiliary subunit of voltage-gated calcium channels) in central nervous system tissues			Focal Aware Onset Seizure	Tiredness, sleepiness
**Primidone**	Mysoline	Unknown, however it has anticonvulsant activity as do its two metabolites, phenobarbital and phenylethylmalonamide (PEMA). In addition to its anticonvulsant activity, PEMA potentiates the anticonvulsant activity of phenobarbital in experimental animals	100 to 125 mg	750 mg	Juvenile Myoclonic Epilepsy<br>Myoclonic Seizures<br>Tonic-clonic Seizures	Unsteadiness<br>Vertigo<br>Irregular eye movements<br>Nausea<br>Vomiting<br>Blurred or double vision<br>Drowsiness<br>Depression
**Rufinamide**	Banzel	Unknown, however *in vitro* studies suggest the modulation of the activity of sodium channels and, in particular, prolongation of the inactive state of the channel	400 to 800 mg	3200 mg	Lennox-Gastaut Syndrome	Headache<br>Dizziness<br>Fatigue<br>Nausea<br>Sleepiness
**Stiripentol**	Diacomit	Unknown, possible mechanisms of action include direct effects mediated through the gamma-aminobutyric acid $GABA_A$ receptor	50 $mg.kg^{-1}$	50 $mg.kg^{-1}$	Dravet Syndrome	Decreased appetite and weight loss<br>Feeling tired or sleepy<br>Low muscle tone, being off balance
**Tiagabine Hydrochloride**	Gabitril	Unknown, however *in vitro* experiments demonstrate the ability to enhance the activity of gamma aminobutyric acid (GABA). This suggests that tiagabine prevents the propagation of neural	4 mg	32 to 56 mg	Focal Impaired Awareness or Complex Partial Seizures<br>Focal Aware Onset Seizure	Dizziness<br>Tiredness<br>Nervousness<br>Sleepiness<br>Difficulty concentrating

		impulses that contribute to seizures by a GABA-ergic action				Tremor
**Topiramate**	Topamax	Unknown, however studies suggest that it blocks voltage-dependent sodium channels, augments the activity of the neurotransmitter gamma-aminobutyrate at some subtypes of the GABA-A receptor, antagonizes the AMPA/kainate subtype of the glutamate receptor, and inhibits the carbonic anhydrase enzyme, particularly isozymes II and IV	25 to 50 mg	400 mg	Lennox-Gastaut Syndrome<br>Focal Impaired Awareness or Complex Partial Seizures<br>Focal Aware Onset Seizure<br>Tonic-clonic Seizures	Fatigue or drowsiness<br>Difficulty with concentration<br>Difficulty finding the right word<br>Confusion<br>Dizziness<br>Unsteadiness<br>A feeling of pins and needles, usually in the tips of the fingers and toes<br>Loss of appetite and weight loss<br>Nervousness<br>Depression<br>Difficulty with memory
**Topiramate XR**	Qudexy XR, Trokendi XR	Unknown, however studies suggest that it blocks voltage-dependent sodium channels, augments the activity of the neurotransmitter gamma-aminobutyrate at some subtypes of the GABA-A receptor, antagonizes the AMPA/kainate subtype of the glutamate receptor, and inhibits the carbonic anhydrase enzyme, particularly isozymes II and IV	50 mg	400 mg	Lennox-Gastaut Syndrome<br>Temporal Lobe Epilepsy<br>Focal Impaired Awareness or Complex Partial Seizures<br>Refractory Seizures<br>Secondarily Generalized Seizures or Bilateral Tonic Clonic Seizure<br>Focal Aware Onset Seizure<br>Tonic-clonic Seizures	Fatigue or drowsiness<br>Difficulty with concentration<br>Difficulty finding the right word<br>Confusion<br>Dizziness

						Unsteadiness<br>A feeling of pins and needles, usually in the tips of the fingers and toes<br>Loss of appetite and weight loss<br>Nervousness<br>Depression<br>Difficulty with memory
**Valproic Acid**	Depakene	Valproic acid dissociates to the valproate ion in the gastrointestinal tract. The mechanisms by which valproate exerts its antiepileptic effects have not been established. It has been suggested that its activity in epilepsy is related to increased brain concentrations of gamma-aminobutyric acid (GABA)	10 to 15 $mg.kg^{-1}$	≤60 $mg.kg^{-1}$	Juvenile Myoclonic Epilepsy<br>Lennox-Gastaut Syndrome<br>Temporal Lobe Epilepsy<br>Focal Impaired Awareness or Complex Partial Seizures<br>Myoclonic Seizures<br>Refractory Seizures<br>Secondarily Generalized Seizures or Bilateral Tonic Clonic Seizure<br>Focal Aware Onset Seizure<br>Tonic-clonic Seizures	Tiredness<br>Dizziness<br>Upset stomach<br>Vomiting<br>Tremor<br>Hair loss<br>Weight gain<br>Changes in behavior
**Fenfuramine**	Sabril	Unknown, but it is believed to be the result of its action as an irreversible inhibitor of γ-aminobutyric acid transaminase (GABA-T). This action results in increased levels of GABA in the central nervous system	1000 mg	3000 mg	Infantile Spasms/West's Syndrome<br>Temporal Lobe Epilepsy<br>Focal Impaired Awareness or Complex Partial Seizures<br>Refractory Seizures<br>Secondarily Generalized Seizures or Bilateral Tonic Clonic Seizure<br>Focal Aware Onset Seizure	Loss of peripheral vision<br>Fatigue<br>Sleepiness<br>Dizziness<br>Tremor<br>Nasopharyngitis<br>Blurry vision

						Memory impairment<br>Stomach upset, constipation or diarrhea
**Zonisamide**	Zonegran, Zonisade	Unknown, however it may the antiseizure effect through action at sodium and calcium channels. *In vitro* pharmacological studies suggest that zonisamide blocks sodium channels and reduces voltage-dependent, transient inward currents (T-type Ca2+ currents),	100 mg	300 to 400 mg	Temporal Lobe Epilepsy<br>Focal Impaired Awareness or Complex Partial Seizures<br>Refractory Seizures<br>Secondarily Generalized Seizures or Bilateral Tonic Clonic Seizure<br>Focal Aware Onset Seizure	Sleepiness or fatigue<br>Dizziness<br>Loss of appetite<br>Upset stomach<br>Headache<br>Agitation or irritability<br>Weight loss
